# Expression and localization of matrix metalloproteinases (MMP-2, -7, -9) and their tissue inhibitors (TIMP-2, -3) in the chicken oviduct during maturation

**DOI:** 10.1007/s00441-015-2290-9

**Published:** 2015-09-22

**Authors:** Agnieszka Leśniak-Walentyn, Anna Hrabia

**Affiliations:** Department of Animal Physiology and Endocrinology, University of Agriculture in Krakow, al. Mickiewicza 24/28, 30-059 Krakow, Poland

**Keywords:** MMPs, TIMPs, Oviduct, Development, Chicken

## Abstract

Although participation of matrix metalloproteinases (MMPs) in reproductive tract remodeling has been strongly suggested in mammalian species, the role of MMPs in the avian oviduct has received little attention. To gain a better understanding of the potential role of the MMP system in avian oviduct development, mRNA and protein expression, localization of selected MMPs and their tissue inhibitors (TIMPs), and gelatinolytic activity in the oviduct of growing chickens were examined. The oviducts were collected from Hy-Line Brown hens before (10, 12, 14 and 16 weeks of age) and after (week 17) the onset of egg laying. The MMP-2, -7, -9 and TIMP-2 and -3 genes were found to be differentially expressed in all examined oviductal sections: the infundibulum, magnum, isthmus and shell gland on both mRNA (by real time polymerase chain reaction) and protein (by western blotting and immunohistochemistry) levels. In the course of oviduct development, the relative expression of all genes decreased in most sections. Protein level of MMP-9 was diminished, while MMP-7 and TIMP-3 were elevated in the oviduct of growing birds. MMP-2 and TIMP-2 protein levels remained constant, with a slight increase in MMP-2 concentration just before reaching maturity. The relative activity of MMP-2 and -9 (assessed by gelatin zymography) was higher (*P* < 0.05, *P* < 0.01) in immature birds compared with adults. Immunohistochemistry demonstrated cell- and tissue-specific localization of MMPs and TIMPs in the wall of the chicken oviduct. We concluded that changes in the expression of examined MMPs and their inhibitors, as well as alterations in MMP activity occurring simultaneously with changes in the morphology of the chicken oviduct, suggest the involvement of the MMP system in the proper development and functioning of this organ. Mechanisms regulating the expression and activity of MMPs require further clarification.

## Introduction

The extracellular matrix (ECM) is one of the most important regulators of cellular physiology, providing an environment for cell migration, proliferation, differentiation, and apoptosis (Smith et al. 1999). Therefore, proper ECM remodeling seems to be essential for tissue growth and morphogenesis. Numerous studies have established that the key enzymes involved in the control of ECM turnover and homeostasis are matrix metalloproteinases (MMPs) (Page-McCaw et al. 2007; Murphy and Nagase 2008; Kessenbrock et al. 2010).

Architectural features and substrate preference categorize the MMP family members into several classes, i.e. the collagenases (MMP-1, -8, -13), gelatinases (MMP-2, -9), stromelysins (MMP-3, -10, -11), matrilysins (MMP-7, -26), membrane type MMPs (MT-MMP-1, -2, -3, -4, -5, -6), and other less characterized members (Page-McCaw et al. 2007). The gelatinases, stromelysins and matrilysins are capable of degrading major constituents of basement membranes, including type IV collagen, laminin and fibronectin (Senior et al. 1991; Nagase et al. 2006). In addition to the removal of unwanted ECM molecules, MMPs exhibit activity toward other MMPs, growth factors and cytokines. It has become clear that MMPs influence many cellular functions and so their activity may be necessary during development and normal physiology (Fingleton 2003; Murphy and Nagase 2008, 2011; Page-McCaw et al. 2007).

MMPs are initially expressed in an enzymatically inactive state and require proteolytic cleavage by convertases, serine proteinases or by other MMPs to become active (Sternlicht and Werb 2001). The site and the extent of ECM remodeling depend on the local balance between the MMPs and their associated endogenous inhibitors. The physiological inhibitors of MMP function are liver-derived α2-macroglobulin and the tissue inhibitors of metalloproteinases (TIMPs). TIMP-1, -2, -3, and -4 act selectively on different MMPs. For example, TIMP-2 has a high affinity for MMP-2, whereas TIMP-3 preferentially binds to MMP-9. Thus, the activity of MMPs can be regulated at multiple levels: gene expression, conversion from zymogen to active enzyme, and, eventually, the presence of specific inhibitors (Murphy 2011).

The MMP system has been strongly suggested to play a critical role in reproductive functions in humans and diverse animal species (Curry and Osteen 2001; Gabler et al. 2001; Bai et al. 2005). It has been postulated to regulate dynamical structural changes that occur in the ovary and uterus. In addition, the expression of MMPs in reproductive tissues has been reported to be cell-, tissue- and reproductive cycle-dependent (Rodgers et al. 1993; Rudolph-Owen et al. 1998; Skinner et al. 1999; Määttä et al. 2000). Although the participation of MMP family members in reproductive functions has been strongly suggested in mammalian species, the evidence regarding MMPs in the avian reproductive system is scarce. It is known that certain MMPs and TIMPs are expressed in the chicken ovary (Leśniak and Hrabia 2012; Zhu et al. 2014), whereas information concerning the MMP system in the avian oviduct is limited to the demonstration of MMP-2, -9 and TIMP-2 and -3 mRNA expression in the chicken (Réhault-Godbert et al. 2008; Dunn et al. 2009; Song et al. 2011; Hrabia et al. 2013), as well as the involvement of MMP-2 in the process of the right Müllerian duct regression in the chicken embryo (Ha et al. 2004).

The avian reproductive system differs from that of mammals, and most of the compartments have widely different anatomical and physiological features. In birds, the oviduct consists of five diverse segments: infundibulum, magnum, isthmus, shell gland (uterus), and vagina, each of which is involved in specific biological events. During development of the oviduct, the epithelial cells differentiate into tubular gland cells, goblet cells and ciliated cells (Kohler et al. 1969). The tubular gland cells are formed in the magnum, isthmus and shell gland, and these oviductal segments have specific secretory functions for egg formation.

Considering the fact that the mechanisms orchestrating avian oviduct development at the molecular level are only poorly understood, the aim of this study was to examine the potential involvement of some members of the MMP system in the process of ECM remodeling in the avian oviduct during puberty. Accordingly, mRNA and protein expression, and localization of selected MMPs (MMP-2, -7, -9) and TIMPs (TIMP-2, -3), as well as gelatinolytic activity in the oviduct of growing chickens were determined.

## Materials and methods

### Experimental birds

Animal experiments were conducted according to research protocols approved by the Local Animal Ethics Committee in Kraków, Poland. Immature Hy-Line Brown chickens (layer strain) were purchased at 9 weeks of age from a commercial farm (Drobeco, Palowice, Poland). Hens were caged individually under a photoperiod of 14 L:10 D with free access to commercial food (11.5 MJ ·kg^−1^, 15 % protein) and water. Birds were killed by decapitation at 10, 12, 14, 16 and 17 weeks of age, i.e., 7, 5, 3 and 1 week(s) before and at the time of reaching maturity, evidenced by the first oviposition (118 ± 1.2 days; *n* = 15 at 10 and 12 weeks, and *n* = 6 at 14, 16 and 17 weeks of age). The hens that laid eggs were decapitated 2 h after oviposition. The oviduct was rapidly isolated and weighed, and these oviductal segments were collected: infundibulum, magnum, isthmus and shell gland. Tissue samples were immediately frozen in liquid nitrogen and kept at –80 °C until determination of the protein expression of MMPs and activity, or placed into RNAlater and stored at –20 °C until total RNA isolation. The other tissue fragments were fixed in freshly prepared 10 % (v/v) buffered (0.1 M phosphate buffer, pH = 7.6) formalin, processed, and embedded in paraffin wax for subsequent localization of MMPs and TIMPs.

### RNA isolation, cDNA synthesis and quantitative (q) PCR

Total RNA was extracted from the tissues using TRI-reagent (MRC, Cincinnati, OH, USA) according to manufacturer’s recommendations. RNA (2 μg from each tissue) was reverse-transcribed with a High-Capacity cDNA Reverse Transcription Kit (Applied Biosystems, Foster City, CA, USA) including random primers. Samples were incubated in a thermocycler (Mastercycler Gradient; Eppendorf, Hamburg, Germany) according to the following thermal profile: 25 °C for 10 min, 37 °C for 120 min and 85 °C for 5 min. The obtained cDNA was used for real-time qPCR for MMP-2, -7 and -9, and TIMP-2 and -3 in a 96-well thermocycler (StepOne Plus; Applied Biosystems) according to the recommended cycling program: 2 min at 50 °C, 10 min at 95 °C, 40 cycles of 15 s at 95 °C and 60 s at 60 °C. The multiplex real-time qPCRs for the examined genes were performed in a 10-μl volume containing 5 μl TaqMan Gene Expression Master Mix (Applied Biosystems), 0.5 μl TaqMan Gene Expression Assays with specific TaqMan MGB-probe and one pair of primers designed by Applied Biosystems, 0.5 μl of Eucaryotic 18S rRNA Endogenous Control (pair of primers and TaqMan probe-labeled VIC/TAMRA as a reference gene; Applied Biosystems), 3 μl water and 1 μl cDNA (10×diluted sample after the reverse transcription). The TaqMan Gene Expression Assay parameters are shown in Table 1. Each sample was run in duplicate. A no-template control was included in each run. Relative quantification of the investigated genes was calculated after normalization with the 18S rRNA transcript, and expression in the infundibulum of the 10-week-old chickens as the calibrator by using the 2^-ΔΔCt^ method. Quantification was performed using StepOne integrated software.

**Table 1:** TaqMan probe sequences and size of amplicons generated by real-time polymerase chain reaction assay for chicken MMP-2, -7 and -9, and TIMP-2 and -3 in the chicken oviduct

Gene	Genbank accession no.	TaqMan probe (FAM5’ → 3’NFQ)	Amplicon (bp)
MMP-2	UO7775.1	CCTGGCCCTGGTCCTG	69
MMP-7	NM_001006278.1	TACAATCAAGGAGTTAATTTGTTCC	92
MMP-9	NM_204667.1	AGTACCTCTATGGTCGTGGCTCTGG	78
TIMP-2	NM_204298.1	ATCGCCCTCGGATTTG	88
TIMP-3	NM_205487.2	ACGCGGCCTGTAATC	70

### Western blot analysis

In order to examine the expression of MMPs at the protein level, immunoblotting was performed. Tissues were homogenized in lysis buffer (BioVision, Milpitas, CA, USA) and clarified by centrifugation (10,000*g*, 5 min, 4 °C). The protein content was estimated using Bradford protein assay (Bio-Rad, Hercules, CA, USA) with bovine serum albumin (BSA) as the standard. Aliquots containing 50 μg of total protein were mixed with loading buffer under reducing conditions and subjected to SDS-PAGE using 12 % polyacrylamide gels (TGX FastCast acrylamide kits; Bio-Rad) and Mini-Protean Tetra Cell apparatus. Resolved proteins were transferred onto nitrocellulose membrane utilizing iBlot Gel Transfer System (Life Technologies, Austin, TX, USA). The membranes were then blocked with 5 % BSA and incubated (overnight at 4 °C) with rabbit anti-MMP-2 (Abcam, Cambridge, UK), rabbit anti-MMP-7 (Santa Cruz Biotechnology, Santa Cruz, CA, USA), rabbit anti-MMP-9 (Abcam), rabbit anti-TIMP-2 (Santa Cruz Biotechnology) or rabbit anti-TIMP-3 polyclonal antibodies (Abcam) at 1:2000 dilution, followed by incubation with goat anti-rabbit HRP-conjugated antibody at 1:10,000 dilution. The bands were visualized using enhanced chemiluminescence (Advansta, CA, USA), ChemiDoc-It 410 imaging system and VisionWorks Life Science software. As an internal control, western blotting with mouse monoclonal anti-β-actin HRP-conjugated IgG (1:500; Santa Cruz Biotechnology) was performed. In negative controls, primary antibodies were omitted.

### Immunohistochemistry

Microtome sections (6 μm thick) were deparaffinized in xylene and rehydrated by passing through graded alcohols. After washing with TBST buffer (Tris buffer saline + 0.1 % Tween 20), slices were incubated in citric buffer (pH = 6.0, 75 °C, 20 min) for heat-induced antigen retrieval. Endogenous peroxidase activity was quenched in 3 % H_2_O_2_ in methanol (10 min). Nonspecific binding of the secondary antibody was blocked by incubation with 5 % (v/v) normal goat serum in TBST (RT, 40 min). Sections were then incubated overnight with primary rabbit polyclonal antibodies against MMPs and TIMPs as described for western blots above. After rinsing with TBST, sections were incubated (1.5 h) with biotinylated goat anti-rabbit antibody (1:300) followed by avidin-biotin-horseradish peroxidase complex (Vector Laboratories, Burlingame, CA, USA) (30 min). The color reaction was developed by incubation with diaminobenzidine (DAB) and H_2_O_2_ solution. Non-specific staining was demonstrated by replacement of the primary antibody with TBST. Slides were examined under a light microscope (Jena Zeiss, Germany).

### Histochemistry

Additionally, for outlining structures such as connective tissue and basement membrane in the wall of the oviduct, the Periodic acid–Schiff’s staining (PAS; Sigma, St. Louis, MO, USA) was performed for 10 min and 15 min, respectively.

### Electrophoretic transfer gelatin zymography

Gelatinolytic activity within oviduct tissue homogenates was determined using transfer zymography as described previously by Pan et al. (2011). Briefly, samples (50 μg of total protein) containing proteolytic enzymes were first resolved in nonreducing SDS–PAGE on a 10 % gel without substrate. The proteins in the resolving gel were then electrophoretically transferred, using Invitrogen gel transfer system, to a previously prepared gel with a copolymerized gelatin (0.5 mg/ml). The receiving gel was then washed 3 times for 15 min in 2.5 % (v/v) Triton X-100 with gentle shaking to remove SDS and incubated at 37 °C overnight in developing buffer (50 mM Tris-HCl, 10 mM CaCl_2_, 200 mM NaCl, 0.05 % NaN_3_, pH 7.5). Subsequently, the gel was stained with 0.5 % (w/v) Coomassie Brilliant Blue R-250 and destained in distilled water until clear bands indicating gelatinolytic activity became visible. An extended-range protein ladder was used for molecular weight estimation of the bands. Negative control consisted of 12 mM 1,10-phenanthroline or 10 mM EDTA (chelates Zn^2+^ and Ca^2+^) added to renaturing and developing buffer before gel incubation.

### Statistical analysis

Data were statistically analyzed by two-way ANOVA followed by Duncan’s multiple range test. Log transformations were performed as needed to maintain homogeneity of variance and normality. For comparison of the means of two groups, the Student’s *t* test was applied. Differences of values were considered to be significant at *P* < 0.05. Calculations were performed with Sigma Stat 2.03 (Systat Software, Germany). Results are presented as means ± SEM.

## Results

### Messenger RNA expression of MMPs and TIMPs in the chicken oviduct during maturation

**Fig. 1:**
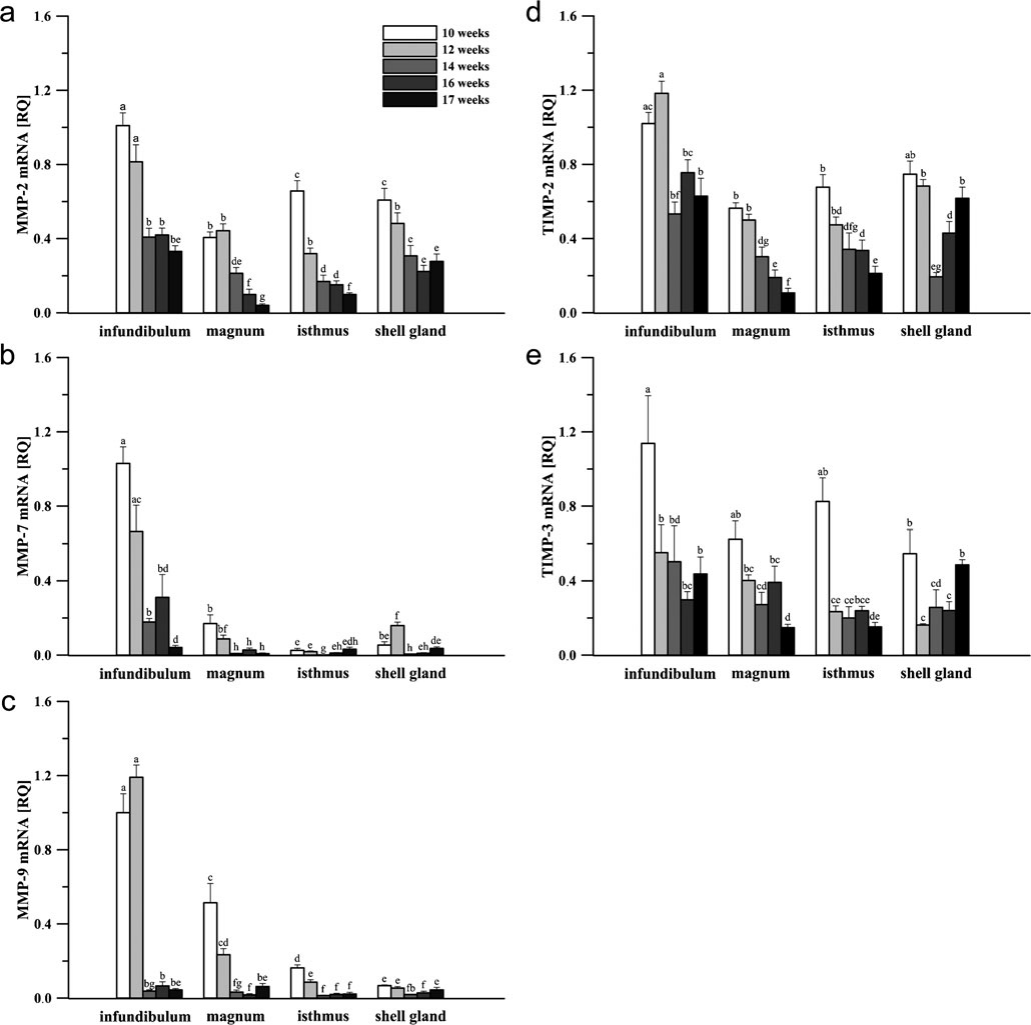
Expression of MMP-2 (**a**), MMP-7 (**b**), MMP-9 (**c**), TIMP-2 (**d**) and TIMP-3 (**e**) mRNAs in the chicken oviduct during maturation. Data represent the mean of relative quantity (RQ) ± SEM from a minimum of six chickens standardized to expression in the infundibulum of 10-week-old birds. Values marked with different *letters* differ significantly (*P* < 0.05)

Each of the studied genes was expressed in the chicken oviduct during sexual maturation. The expression of MMP system members was found to be age- and oviductal section-dependent. The relative expression (RQ) of MMP-2, -7, -9 and TIMP-2 and -3 mRNA is shown in Fig. 1a-e.

As shown in Fig. 1a, the expression of MMP-2 was highest in the infundibulum and lowest in the magnum. A relatively high level of MMP-2 mRNA noted in the oviductal segments in 10-week-old chickens decreased (*P* < 0.05) in the isthmus and shell gland by 51 and 21 %, respectively. At the age of 14 weeks, expression of MMP-2 declined (*P* < 0.05) in all segments (infundibulum by 50 %, magnum by 52 %, isthmus by 47 %, and shell gland by 36 %). At the following stage of the development (16 weeks), the expression of MMP-2 was down-regulated only in the magnum (by 54 %) when compared to the 14-week-old chicks. Finally, magnum and isthmus of 17-week-old laying hens showed lower (*P* < 0.05) MMP-2 mRNA levels (by 60 and 35 %) than that of chickens at 16 weeks.

The expression of MMP-7 in the developing oviduct was most abundant in the infundibulum, whereas it was lowest in the isthmus (Fig. 1b). Between the ages of 10 and 12 weeks, an increase (*P* < 0.01) in the MMP-7 expression was noted in the shell gland (by 194 %), while the expression in other oviductal parts tended to decrease. In birds between 12 and 14 weeks of age, the mRNA level diminished (*P* < 0.01) substantially in all of the examined sections: the infundibulum (by 73 %), magnum (by 91 %), isthmus (by 94 %) and shell gland (by 96 %), and became barely expressed. In 16-week-old hens, the expression of MMP-7 mRNA was raised (*P* < 0.001) in the isthmus by 900 %. In mature birds, the level of MMP-7 mRNA remained unchanged (Fig. 1b).

The highest relative expression of MMP-9 was detected in the infundibulum and the lowest in the shell gland (Fig. 1c). Between 10 and 12 weeks of age (*P* < 0.05), a decrease in the MMP-9 mRNA level was observed in the isthmus (by 47 %). As in other MMPs tested, a tremendous reduction (*P* < 0.05; *P* < 0.01) in the expression of MMP-9 was noted between 12 and 14 weeks in all the sections: the infundibulum (by 97 %), magnum (by 86 %), isthmus (by 84 %), and shell gland (by 65 %). At the following stage of development (16 weeks), the expression of MMP-9 did not vary when compared to the 14-week-old chicks, although in laying hens an increase (*P* < 0.01; *P* < 0.05) in the magnum and shell gland was observed, by 255 and 57 %, respectively (Fig. 1c).

TIMP-2 showed a similar expression profile to MMP-2, with the highest mRNA level in the infundibulum and the lowest in the magnum (Fig. 1d). The highest relative expression of TIMP-2 was revealed at the ages of 10 and 12 weeks, and substantially declined (*P* < 0.05; *P* < 0.01) at week 14 in the infundibulum, magnum and shell gland by 55, 40, and 72 %, respectively. What is interesting is that the TIMP-2 mRNA level reduced (*P* < 0.05) in the magnum of 16-week-old chicks by 37 %, while it increased (*P* < 0.01) in the shell gland by 121 %. Similarly, in laying hens, the expression of TIMP-2 dropped (*P* < 0.05) in the magnum and isthmus (by 44 and 37 %), whereas it was up-regulated (*P* < 0.05) in the shell gland (by 47 %) (Fig. 1d).

The highest relative expression of TIMP-3 was detected in the infundibulum of pullets at 10 weeks and the lowest was observed in the magnum and isthmus of 17-week-old hens (Fig. 1e). A meaningful drop (*P* < 0.05) of the TIMP-3 mRNA was detected between 10 and 12 weeks of age in the infundibulum by 51 %, isthmus by 72 %, and shell gland by 70 %. At the following stages of development, the expression did not vary substantially (*P* > 0.05) until 17 weeks, when it declined (*P* < 0.05) in the magnum by 62 % and rose in the shell gland by 103 % (Fig. 1e).

### Protein expression of MMPs and TIMPs in the chicken oviduct during maturation

**Fig. 2:**
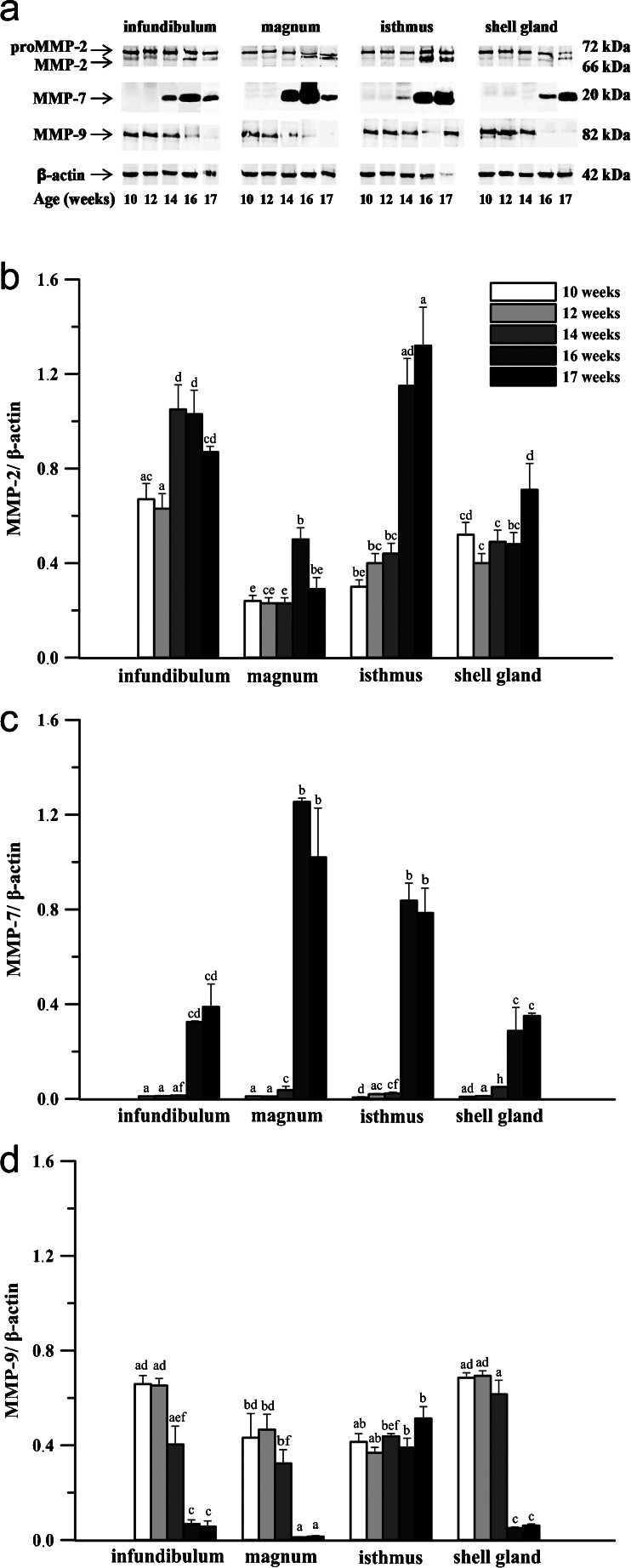
Western blot analysis of MMP-2, -7 and -9 proteins in the chicken oviduct during maturation. **a** Representative blots of a minimum of 3 independent determinations. **b**–**d** Densitometric analysis for MMPs. The relative expression of each protein was evaluated with densitometry and expressed as the ratio relative to β-actin. Each value represents the mean ± SEM from a minimum of 3 determinations; values marked with different *superscript letters* differ significantly (*P* < 0.05)

**Fig. 3:**
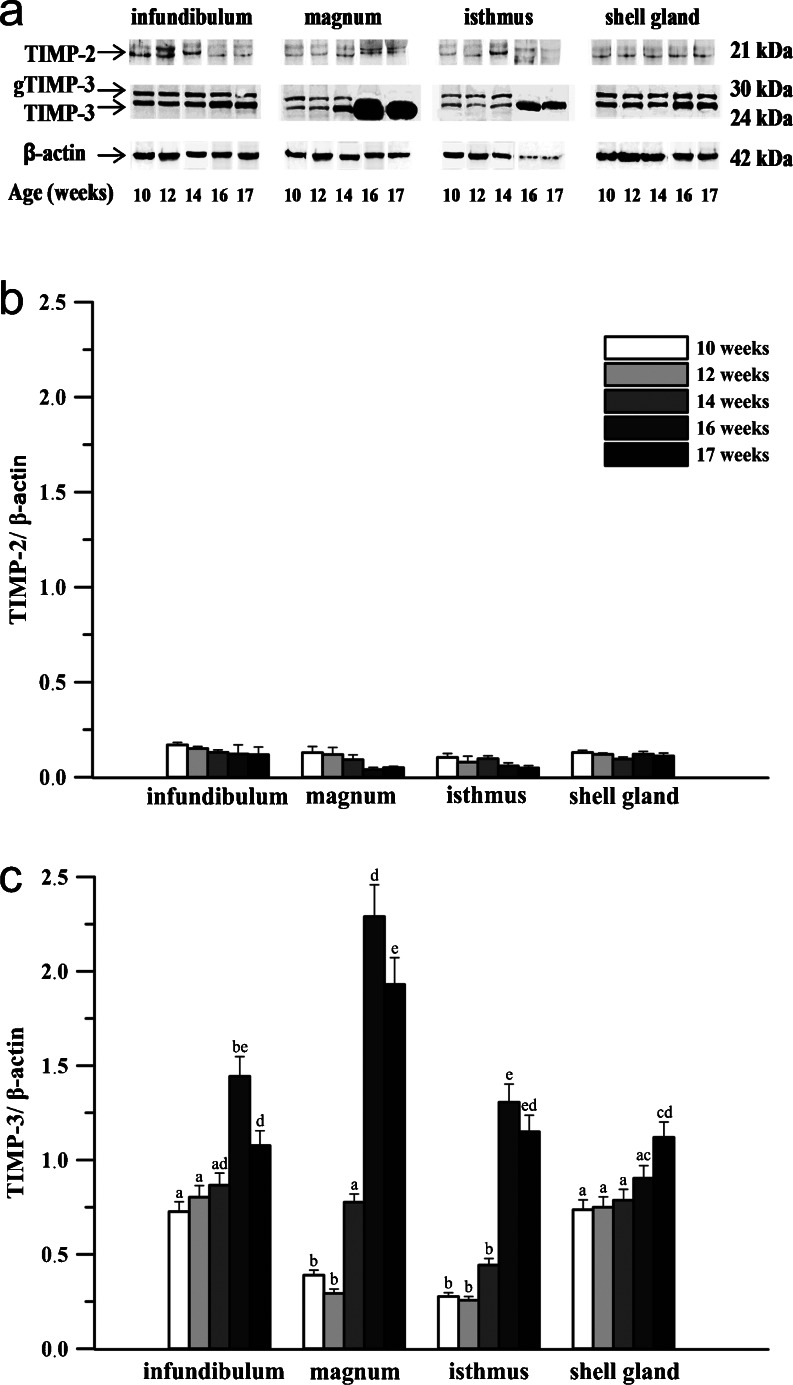
Western blot analysis of TIMP-2 and -3 proteins in the chicken oviduct during maturation. **a** Representative blots of a minimum of 3 independent determinations. **b**, **c** Densitometric analysis for TIMPs. The relative expression of each protein was evaluated with densitometry and expressed as the ratio relative to β-actin. Each value represents the mean ± SEM from a minimum of 3 determinations; values marked with different *superscript letters* differ significantly (*P* < 0.05)

The developmental changes in MMP-2, -7, -9 and TIMP-2 and -3 protein levels in the chicken oviduct were detected by western blot. All examined elements of MMP system were identified in oviductal tissues, and their amount was dependent on oviductal segments and the age of birds (Figs. 2a, 3a). At each developmental stage, both latent (72 kDa) and active (66 kDa) forms of MMP-2 were detected (Fig. 2a). The highest relative level (sum of both forms) was in the infundibulum and isthmus, and the lowest in the magnum. Moderate expression of protein observed in the oviduct of 10- and 12-week-old chickens increased (*P* < 0.05; *P* < 0.01) in the infundibulum at the age of 14 weeks by 65 %, and at 16 weeks in the magnum and isthmus by 115 and 164 %, respectively. In laying hens, an elevation of protein level by 49 % was found in the shell gland (Fig. 2b). Moreover, in chickens at the age of 16 and 17 weeks, an increase in active form of MMP-2 was observed, especially in the isthmus (Fig. 2a).

During maturation, the presence of MMP-7 protein, mainly its active form (~20 kDa), was found in the chicken oviduct (Fig. 2a). In several samples of the magnum and isthmus, an additional band (~32 kDa) corresponding to the latent form of MMP-7 was also observed. The highest MMP-7 protein level was detected in the magnum and the lowest in the infundibulum and shell gland. Very low levels of MMP-7 detected in the oviduct of 10- and 12-week-old birds increased (3-fold) in the magnum and shell gland of 14-week-old hens. The most pronounced increase (*P* < 0.001) in protein expression occurred in 16-week-old birds in the infundibulum (22-fold), magnum (33-fold), isthmus (35-fold) and shell gland (5-fold). After maturation, there was no change in MMP-7 protein expression (Fig. 2a, c).

MMP-9 protein was found mainly in active form (~82 kDa) in the oviduct of growing chickens (Fig. 2a). Between 10 and 14 weeks of age, the relative levels of MMP-9 protein were relatively high in all parts of the oviduct, and at the following stage decreased (*P* < 0.01) in the infundibulum by 83 %, magnum by 96 % , and shell gland by 92 % (Fig. 2d).

TIMP-2 protein (~21 kDa) was also detected in chicken oviduct during maturation (Fig. 3a). It was present at relatively low levels in all segments of the oviduct and did not change (*P* > 0.05) during the examined period (Fig. 3b).

TIMP-3 protein was found in glycosylated (~30 kDa) and unglycosylated (~24 kDa) forms (Fig. 3a). At the age of 10 weeks, the moderate expression of TIMP-3 protein was observed, and it increased (*P* < 0.05) at the age of 14 weeks in the magnum by 156 %, and markedly increased (*P* < 0.01) at the age of 16 weeks in the infundibulum, magnum and isthmus by 66, 200 and 195 %, respectively (Fig. 3c). After maturation, TIMP-3 protein levels decreased (*P* < 0.05) in the infundibulum by 25 % and magnum by 16 %. In 16- and 17-week-old chickens, the unglycosylated form of TIMP-3 was mainly detected, and seen especially in the magnum and isthmus which were characterized by the highest levels of TIMP-3.

### PAS staining and immunohistochemical localization of MMPs and TIMPs in the chicken oviduct during maturation

A slight increase in oviductal weight (g) was observed from 10 to 14 weeks of age (0.12 ± 0.01 → 2.22 ± 1.06) followed by a sharp increase at 16 weeks (30.3 ± 6.78). At 17 weeks, i.e., at the time of sexual maturity, the weight of the oviduct was 50.5 ± 4.29.

**Fig. 4:**
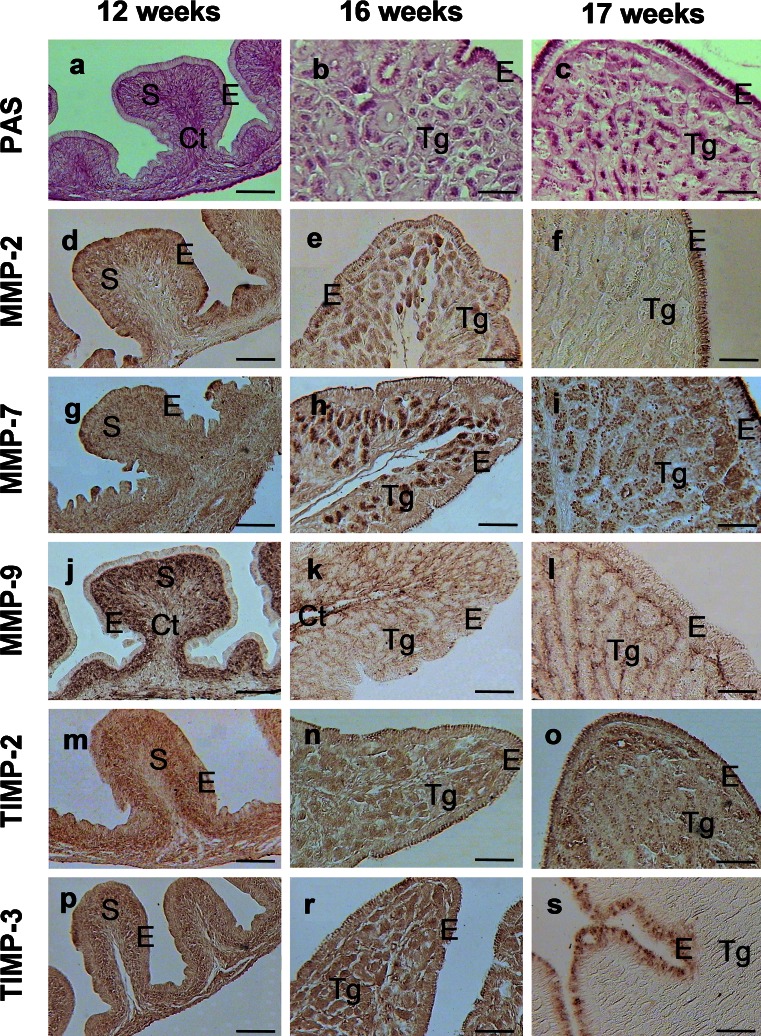
PAS staining (**a**–**c**) and immunohistochemical localization of MMPs (**d**–**l**) and TIMPs (**m**–**s**) in the wall of the oviductal magnum during sexual maturation of chicken. **a** PAS staining showed connective tissue (*Ct*) and **b**, **c** substances produced by the cells of epithelium (*E*) and tubular glands (*Tg*). **d**, **g**, **m**, **p** MMP-2, -7, TIMP-2 and -3 were present in the epithelium and stroma (*S*) of 12-week-old birds, whereas **j** MMP-9 was localized in external part of stroma. **e**, **h**, **n**, **r** Examined elements of MMP system were found in the epithelium and developing tubular glands of 16-week-old chickens, except **k** MMP-9 in which localization was limited to connective tissue. **f**, **s** In 17-week-old laying hens, MMP-2 and TIMP-3 were present in the epithelium, while **i**, **o** MMP-7 and TIMP-2 were present in the epithelium and tubular glands. **l** MMP-9 was localized only in the connective tissue. *Bars* 100 μm

Developmental changes which occurred in the structure of the wall of the chicken oviduct are demonstrated in Fig. 4a–c. The magnum was shown as a representative segment of the oviduct. Since between 10 and 14 weeks of age, the changes in the wall of the oviduct were very slight; the micrographs from birds at the age of 12 weeks were chosen as an example. The wall of immature birds consisted of poorly differentiated luminal epithelium, the stroma under epithelium and layer of smooth muscles interspersed with connective tissue (Fig. 4a). In chickens at the age of 16 weeks, differentiating cells of tubular glands and epithelium were observed (Fig. 4b), and in 17-week-old birds (matured), all structures characteristic of a fully developed organ were present (Fig. 4c).

In immature 10- to 14-week-old birds, PAS staining allowed observation of the connective tissue including the base membrane (Fig. 4a), and, in 16- and 17-week-old hens, additional intense staining was present in the apical part of the epithelium and in the cells of tubular glands, which may indicate a significant amount of proteoglycans synthesized and secreted by epithelial and tubular gland cells (Fig. 4b, c). Moreover, a different intensity of PAS staining in connective tissue of particular segments of the oviduct was observed which may indicate differences in this tissue composition dependent on the oviductal part.

Specific immunoreactivity for MMPs and TIMPs was found in the wall of all segments of the growing chicken oviduct. Localization of MMPs and TIMPs is demonstrated in the magnum as an example (Fig. 4d–s). Distinct cell- and tissue-specific patterns of the localization of MMPs and TIMPs were observed during puberty.

The intensity of staining for MMP-2 and TIMP-3 proteins in immature birds was highest in the mucosa, but in laying hens it was present only in the mucosal epithelium. Moderate immunopositive reaction for MMP-7 and TIMP-2 was observed in the wall of the juvenile oviduct, whereas a very strong staining, especially for MMP-7, appeared in the developing tubular glands of 16-week-old chickens, as well as apically in mucosal epithelium of mature 17-week-old birds. In immature chicks, the MMP-9 was localized in a large amount in the mucosa except for the mucosal epithelium, while in 16-week-old and adult hens MMP-9 was clearly present only in the connective tissue.

### Gelatinolytic activity of MMPs in the chicken oviduct during maturation

**Fig. 5:**
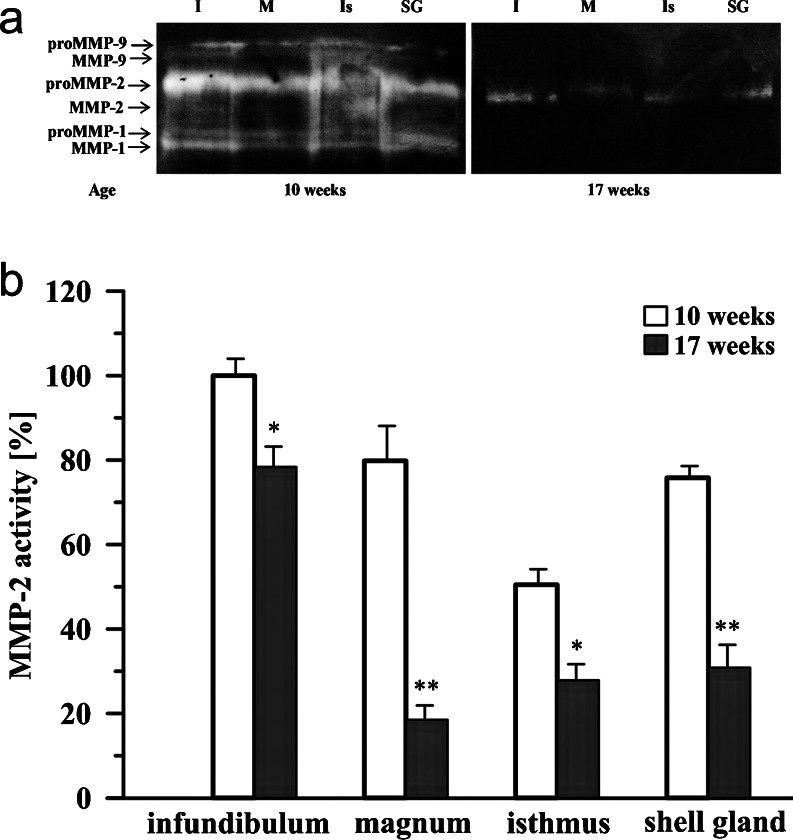
Gelatinase activity in the oviduct of chickens at the age of 10 and 17 weeks. **a** Representative zymograms. *I* infundibulum, *M* magnum, *Is* isthmus, *SG* shell gland. **b** Densitometric analysis for MMP-2 activity. Each value represents the mean of relative activity (%) ± SEM from a minimum of 3 determinations standardized to control activity, which was set at 100 %, in the infundibulum of 10-week-old chickens. *P < 0.05, **P < 0.01 compared with 10-week-old birds

Gelatinase zymography detected latent (~72 kDa) and active (~66 kDa) forms of MMP-2, both in immature and mature chickens (Fig. 5a). During maturation, the activity of MMP-2 decreased (*P* < 0.05, *P* < 0.01). In the oviduct of laying hens (17-week-old) the relative activity of MMP-2 was lower than in 10-week-old chickens: in the infundibulum by 22 %, in the magnum by 77 %, in the isthmus by 46 %, and in the shell gland by 63 % (Fig. 5b).

Zymography also showed the presence of latent (~94 kDa) and active (~82 kDa) forms of MMP-9 in the oviduct of 10-week-old pullets, whereas in 17-week-old hens, activity of MMP-9 was under the sensitivity of the method (Fig. 5a). Moreover, in immature chickens, the presence of two bands of approximately 60 and 50 kDa was detected, which suggests latent and active forms of MMP-1 (Fig. 5a).

## Discussion

The present study demonstrates, for the first time, the expression of MMP-2, -7 -9 and TIMP-2 and -3 at mRNA and protein levels as well as activity of MMP-2 and -9 in particular segments of the chicken oviduct during maturation. Existing data related to MMPs were fragmentary and revealed only the presence of MMP-2, -9 and TIMP-2 and -3 transcripts in some parts of the chicken oviduct (Réhault-Godbert et al. 2008; Dunn et al. 2009; Song et al. 2011; Hrabia et al. 2013).

In general, several weeks before sexual maturity, using the method of real-time PCR, the highest expression level of examined MMPs and TIMPs was detected in 10-week-old chickens and it tended to decrease during the maturation period. MMP-2 and TIMP-2 and -3 demonstrated a similar expression pattern with the most eminent drop of the mRNA expression between 12 and 14 weeks, or 10 and 12 weeks in the case of TIMP-3. A distinct profile of the expression was observed for MMP-7 and -9, with barely detectable mRNAs in the oviduct of 14-week-old and older chickens. Previously, Song et al. (2011) had found that, among genes in which expression changed during growth, development and differentiation of the oviduct, were MMP-2 and -9. They observed that the MMP-2 mRNA level decreased during maturation, whereas MMP-9 increased, but only in birds with a partly differentiated oviduct. Similarly, in our study, levels of MMP-2 and -9 transcripts decreased in all oviductal parts between 10 and 14 weeks of age. In mature birds, expression of MMP-9 mRNA increased in the magnum and shell gland. Dunn et al. (2009) examined transcriptional profiles of genes in the shell gland of immature (12-week-old) and mature (25-week-old) chickens and found that, among 266 genes, TIMP-3 was showing differentiating expression and its mRNA expression was higher in adult birds. Similar observations were noticed in the current study when comparing levels of transcripts in the shell gland of 12- and 17-week-old birds. Also, in the uterus of mice, the differences in MMP and TIMP transcript levels were revealed during embryonic and postnatal development (Hu et al. 2004; Nuttall et al. 2004). The expression of MMP-2 and TIMP-3 in the uterus was relatively high, inhibitor TIMP-2 showed moderate expression, and the presence of the transcripts for MMP-7 and -9 was negligible. The results obtained in this investigation similarly show a very low expression of MMP-7 and -9 in most sections of the oviduct.

Another important observation was the determination of differential expression of MMPs and their inhibitors among oviductal sections, which is in accordance with the finding of Réhault-Godbert et al. (2008), who also showed differences in the level of mRNA expression of MMP-2 and TIMP-2 and -3 among particular segments of the oviduct of the laying hen. These differences may result from the specific structure, cell populations and functions of the various segments of the oviduct. Relatively high expression in the infundibulum can be associated with a large proportion of connective tissue and muscles in this section, which were revealed to be characterized by a high level of transcription of elements of the MMP system (Hu et al. 2004; Nuttall et al. 2004). Furthermore, the relatively low level of transcripts in the magnum, at least in part, may be due to the transcription process being dominated by the genes encoding the proteins included in the egg white.

In this study, the expression of all tested components of the MMP system at the protein level was also shown in the chicken oviduct during maturation. In the case of MMP-7 and TIMP-3, a significant increase in protein level was observed in 16-week-old birds, which may be related to the differentiation of tubular gland cells that appeared at this stage of oviduct development. Of particular interest in this work, the MMP-2, -7 and TIMP-3 protein levels increased in most of the sections of the oviduct during maturation in contrast to expression at the mRNA level. A discrepancy between the expression level of the protein and mRNA could be caused, at least in part, by posttranscriptional regulatory mechanisms. Over the past several years, it has been demonstrated that the expression of genes and organ development, including the reproductive ones (Sirotkin et al. 2009, 2010; Nothnick 2012), are largely regulated by micro-RNAs (mi-RNAs). The inability of the female to maintain pregnancy has been associated with the lack of molecules of *mi-17-5p* and *let7b*, which control the expression of the inhibitor TIMP-1 (Otsuka et al. 2008). In other studies, the ability of *miR-29* to inhibit the expression of several types of collagen and to stimulate the expression of MMP-2 and -9 has been observed (Chen et al. 2011). The possibility cannot be excluded that the molecules of mi-RNA affect the posttranscriptional regulation of MMPs in the tissues of the oviduct in the chickens during sexual maturation. However, despite the discovery of several mi-RNAs involved in the mechanisms regulating growth and differentiation of the hen oviduct (Jeong et al. 2012), there are as yet no identified molecules which are capable of affecting the expression of MMPs in this organ in birds.

The method of immunodetection and zymography confirmed the presence of both forms of MMP-2, latent and active, in the oviduct of growing chickens. Expression of MMP-2 at the protein level increased during maturation while the activity of this metalloproteinase decreased in the oviduct. This may indicate the existence of mechanisms that regulate the activity of this enzyme in the chicken oviduct. One reason for the reduced activity of MMP-2 could be an insufficient amount of inhibitor TIMP-2, the protein level of which was not changed during the examined period. TIMP-2 is essential, not only for inhibition but also for the activation of pro-MMP-2 (Wang et al. 2000; Cantemir et al. 2004). Similarly, activation of MMP-2 is carried out with TIMP-3 (Zhao et al. 2004), and its expression at the protein level increased significantly during the development of the hen oviduct. It cannot be ruled out that other factors regulate the activity of MMP-2, such as claudins, the presence of which has been found in the avian oviduct (Ariyadi et al. 2013) and α2-macroglobulin. Lim et al. (2011) found a high mRNA expression of α2-macroglobulin in the hen oviduct, especially in the tubular glands and epithelium, while it was absent in other examined organs such as the brain, heart and ovaries. Among the sections of oviduct, the greatest expression of this non-specific protease inhibitor was observed in the magnum, and to a lesser extent in the isthmus and the shell gland. A high level of α2-macroglobulin expression in those sections may indicate its role in controlling proteolytic activity (Nagase and Harris 1983). Both protein expression and activity of MMP-9 declined markedly in the oviduct during maturation of chickens. A significant amount of MMP-9 present between 10 and 14 weeks of age strongly suggests its participation in the processes occurring at the early stages of the oviduct development. Previously, Hu et al. (2004) revealed the presence and activity of MMP-2 and -9 in the mouse uterus during development and, similarly, as in the present study in the chicken oviduct, observed mostly the active form of MMP-9.

Subsequently, cell-specific and tissue-specific localization of examined components of the MMP system was observed in the wall of the oviduct. MMP-9 was located predominantly in the connective tissue, while MMP-2 was in the stroma as well as in epithelial and glandular cells. These cells may therefore be responsible for the synthesis and secretion of MMP-2 to the lumen of the oviduct. MMP-2 may then become one of the components of the egg white, since the presence of pro-MMP-2 has been found in both the oviductal fluid and egg white (Réhault-Godbert et al. 2008). The authors identified a pro-MMP-2 in the egg protein in a complex with an inhibitor TIMP-2, although the physiological role of this enzyme is still unclear. The presence of MMP-7 was exclusively found in the cells of epithelium and tubular glands. Inhibitors TIMP-2 and -3 were located in the epithelium and in a small amount in the stroma. Furthermore, the intensity of immunoreactivity for the tested elements of the MMP decreased with the age of the chickens, with the exception of MMP-7, for which the intensity of the reaction increased during maturation. Similarly, Hu et al. (2004) demonstrated cell- and tissue-specific localization of MMPs in the wall of the mouse uterus during its development. In turn, Huh and Jung (2013) observed a decrease in the intensity of immunohistochemical reaction for TIMP-2 in the testes of cocks during puberty.

Changes in the expression and activity of MMPs in the chicken oviduct during sexual maturation strongly indicate that they participate in the processes occurring during the growth and differentiation of this organ. Inherent in tissue morphogenesis is the reconstruction of the components of the ECM, a metalloproteinase domain. Thus, these enzymes can be essential for the migration of cells through the degradation of ECM components, for changes in the behavior of cells by modification of their microenvironment, and for modulation of molecule activity. It has been shown that inhibition of MMP-2 minimizes the migration of adipocytes and the ability to organize in three-dimensional structures (Brown et al. 1997). The lack of activity of MMP-2 also prevents the formation of islets of Langerhans by endothelial cells, without affecting their differentiation (Miralles et al. 1998). So one of the aspects of MMP-2 during development of the chicken oviduct could be its participation in the process of tubular gland formation. This suggestion is supported by its localization in the cells of glandular mucosa of 16-week-old birds. Furthermore, Vaillant et al. (2003) observed that blocking of the MMP-9 function by using a specific antibody reduces cell migration in the developing cerebellum and reduces their susceptibility to apoptosis. Moreover, MMP-9 and TIMP-3 were shown as the key regulators of the degradation of ECM in the uterus of mouse embryos during implantation (Whiteside et al. 2001). It has also been shown that blocking of MMP-9 inhibits the development of renal glomerules and inhibition of the expression of TIMP-2 stimulates the process (Lelongt et al. 1997). Intense immunoreactivity for MMP-9 observed in the stroma adjacent to the epithelium in pullets between 10 and 14 weeks of age may suggest its involvement in the degradation of ECM and the formation of the mucosa in the hen oviduct. Co-localization of MMP-9 with TIMP-2 and -3 may indicate the role of these inhibitors in the control of MMP-9 activity, and in consequence the location and extent of tissue remodeling. The observed increase in MMP-7 expression at the protein level and its localization in the tubular glands of 16- and 17-week-old birds may be associated with oviductal development. Previous studies have shown that mice deficient in the CD44 antigen, which is a receptor docking MMP-7 on the cell surface, have breast abnormalities resulting from the wrong location of the enzyme (Murphy and Nagase 2011; Bonnans et al. 2014). This observation emphasizes the great importance not only of the appropriate level of expression of MMPs but also their location.

Metalloproteinases may also largely determine the activity of biologically relevant molecules by disconnecting them from binding proteins or releasing them from the places of storage in the ECM and thus affecting the development of the avian oviduct. For example, digestion of decorin by MMP-2 and -7 releases the associated transforming growth factor β (TGFβ; Imai et al. 1997), which in turn controls the proliferation, differentiation and apoptosis of most cell types (Conery et al. 2004; Elliott and Blobe 2005). In the chicken, several isoforms of TGFβ and their receptors have been identified (Chowdhury et al. 2003, 2004) and participation of TGFβ in oviductal growth, differentiation and morphogenesis as well as immunosuppression has been suggested (Huang and Huang 2005; Das et al. 2006). Hence, the involvement of MMPs in the development of the oviduct of birds may be associated with the regulation of the availability of TGFs.

Through release from binding proteins, MMPs can also regulate the bioavailability of growth factors such as insulin-like growth factors (IGFs) which influence the tissue growth. It has been found that MMP-2 has activity against insulin-like growth factor-binding proteins 3 and 5 (IGFBP-3 and -5) (Fowlkes et al. 1994; Thrailkill et al. 1995), while both gelatinases are capable of proteolytic activation of TGFβ and IL-1β (Yu and Stamenkovic 2000). Recent studies by Yu et al. (2015) showed that IGF-I stimulates proliferation of myoblasts and development of muscles in chicken embryos. During the development of the quail oviduct, Kida et al. (1994) observed that high expression of IGF-I was accompanied by intensification of DNA replication. In vitro experiments have also shown that IGF-I stimulates the synthesis of ovalbumin in the quail oviduct (Kida et al. 1995). Therefore, it cannot be ruled out that the role of MMPs in the oviduct of birds is associated with the regulation of the activity of IGFs.

In summary, reported changes in the expression of examined MMPs and their inhibitors, as well as alterations in MMP activity occurring simultaneously with changes in the morphology of the chicken oviduct, suggest the involvement of the MMP system in the proper development and functioning of this organ. Mechanisms regulating the expression and activity of MMPs require further clarification.
